# Effects of physical therapy on lung function in children with asthma: Study protocol for a systematic review and meta-analysis

**DOI:** 10.1097/MD.0000000000015226

**Published:** 2019-04-12

**Authors:** Qiu Wang, Weijian Zhang, Lilong Liu, Wenhao Yang, Hanmin Liu

**Affiliations:** a Department of Pediatrics, West China Second University Hospital, Sichuan University, Chengdu; b Department of Gastrointestinal Surgery, People's Hospital of Deyang City, Deyang, Sichuan, China.

**Keywords:** asthma, children, lung function, physical therapy

## Abstract

**Background:**

Morbidity of asthma in children is increasing, which is significantly affecting children's life quality. Despite the medication therapy, physical therapies, including breathing exercises, inspiratory muscle training and physical training, are widely used to improve children's condition. However, the effectiveness of physical therapy remains unclear. This systematic review and meta-analysis is aiming to evaluate the effects of physical therapy on lung function in children with asthma and to assess which physical therapy is more effective.

**Methods:**

Three main databases (PubMed, Embase, and the Cochrane Library) will be searched from inception to November 30, 2018 for randomized controlled trials investigating the effects of physical therapy on lung function in children (age < 18 years old) with asthma published in English. In addition, a manual search of the references of relevant published studies in English will also be considered.

Two independent reviewers will conduct studies selection, data extraction, and risk of bias assessment. Outcome measures will be the Peak Expiratory Flow (PEF), the Forced Expiratory Volume in the first second (FEV1), and the Forced Vital Capacity (FVC). Subgroup analyses will be performed according to the physical therapy (breathing exercises, inspiratory muscle training, and physical training) and the outcome (PEF, FEV1, FVC).

**Results:**

The results will provide useful information about the effect of physical therapy on lung function in children with asthma and demonstrate which physical therapy is more effective.

**Conclusion:**

The findings of this study will be published in a peer-reviewed journal.

**Prospero registration number:**

CRD42019121627

## 1 Introduction

Asthma is one of the most common chronic respiratory diseases in children all over the world, significantly affecting children's health and life quality.^[1]^ Although the prevalence of asthma in children among some high-income countries started to decrease, many low- and middle-income countries with large populations showed increases in prevalence, leading to a great significant burden on worldwide healthcare system.^[2]^ Etiology of asthma is still not clear, but the causes are often discussed on genetic factors and environmental factors.^[3]^ Well-known environment factors include virus infection,^[4,5]^ smoke exposure,^[6,7]^ particulate matter exposure,^[8,9]^ and ozone exposure^[10,11]^ etc. Although GATA3, KIAA1109, and MUC5AC were identified as significant with asthma,^[12]^ the specific genetic factor remains to be clarified.

Medication therapy has been used to control asthma for a long time.^[13–15]^ Despite the medication, physical therapy is another important treatment for children with asthma and widely used in the globe. The main physical therapies for asthma are breathing exercises, inspiratory muscle training, and physical training.^[16–18]^

However, whether the physical therapy improves the lung function in children with asthma is still unclear. Furthermore, there is no systematic review and meta-analysis including all these 3 main physical therapies to evaluate the lung function in children with asthma. In this study, we are aiming to perform a systematic review and meta-analysis to investigate the effects of physical therapy on lung function in children with asthma and demonstrate which physical therapy is more effective.

## 2 Methods

### 2.1 Registration

This study protocol has been registered in the PROSPERO and the registration number is CRD42019121627. The *Cochrane Handbook for Systematic Reviews of Interventions* will be used as a guideline^[19]^ and the software RevMan 5.3 will be used to construct the meta-analysis. We will report this study in accordance with the PRISMA statement also.^[20]^ No ethical statement will be required for this study because there is no direct involvement of human.

### 2.2 Eligibility criteria

#### 2.2.1 Types of studies

Randomized controlled trials (RCTs) published in English up to November 30, 2018 will be included. Trials without well-described randomization methods or those with quasi-random allocation will be excluded.

#### 2.2.2 Types of participants

We will include participants aged <18 years old irrespective of gender and ethnicity. Those including participants aged ≥18 years old will be excluded. All participants must be diagnosed as asthmas by clearly defined or internationally recognized criteria.

#### 2.2.3 Types of interventions

Intervention with physical therapy for asthma could be breathing exercises, inspiratory muscle training or physical training. Those reported with pharmacological, psychological, or behavioral interventions will not be considered. Intervention should be performed with a minimum duration of 2 weeks.

#### 2.2.4 Types of outcome measures

Lung function will be compared between the group with intervention and the group with no intervention. To measure lung function, one of the following outcomes should be reported: the Peak Expiratory Flow (PEF), the Forced Expiratory Volume in the first second (FEV1), and the Forced Vital Capacity (FVC).

### 2.3 Search methods

We will search the three main databases from their inception to November 30, 2018: PubMed, Embase, and the Cochrane Library. The search strategy will involve terms including *child*, *asthma*, *physical therapy,* and *RCT*. A preliminary search strategy in PubMed is described in Table 1. Same search strategy will be used in Embase and the Cochrane Library based on different specific requirements. We will also scan the reference lists of studies and relevant systematic reviews for additional trials.

**Table 1:**
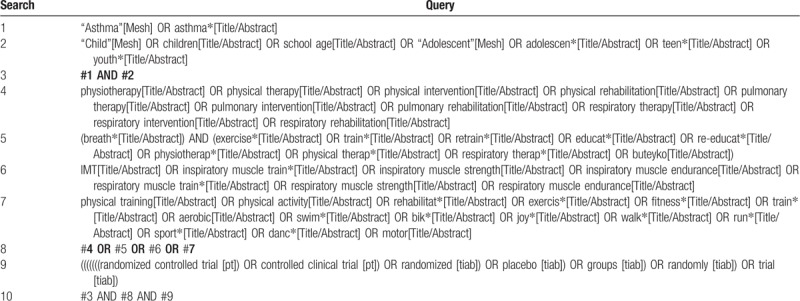
Preliminary search strategy in PubMed.

### 2.4 Study selection and data extraction

#### 2.4.1 Study selection

**Figure 1:**
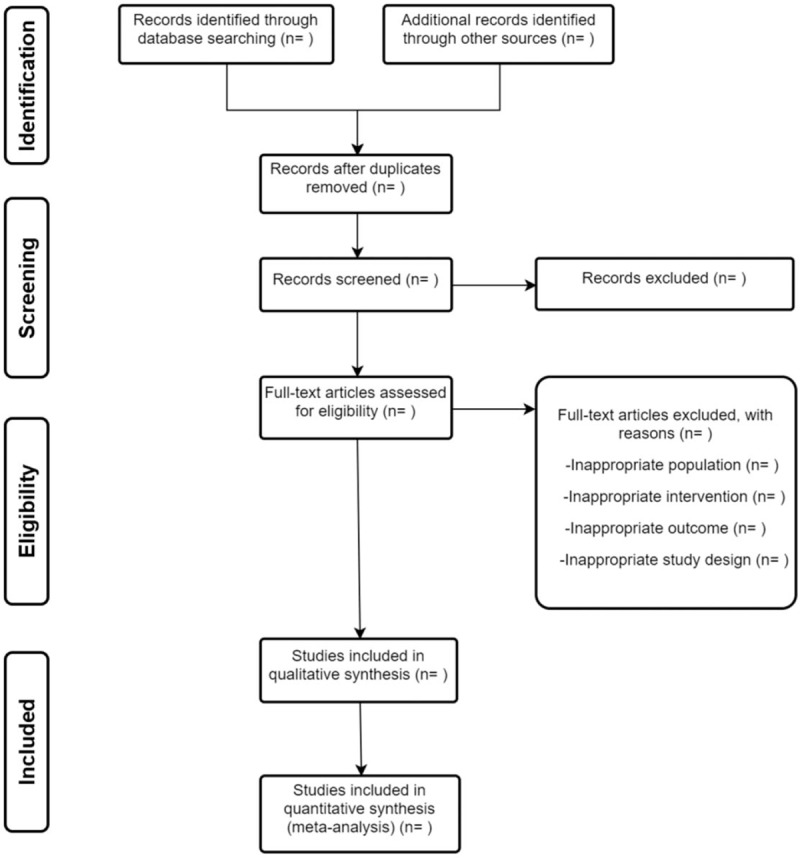
Flow diagram of study selection.

Study selection will be performed by two authors independently. The search results from three electronic databases and additional trials from other resources will be sent to Endnote. We will screen the records after duplicates removed following the two steps: (1) by reading the title and abstract, (2) by reading the full texts. Whether a study will be included is based on the eligibility criteria mentioned above. The reason for studies excluded on full text should be noted. Any different opinions between two authors should consent with the help of a third author. The process of study selection is summarized in a PRISMA flow diagram (Fig. 1).

#### 2.4.2 Data extraction

Two authors will also perform data extraction independently by using a standardized form. The contents will include: generation information of the study (title, author, year, country, etc.), population (number, baseline characteristics, diagnosis criteria, etc.), intervention (type of physical therapy, duration, etc.), outcomes (PEF, FEV1, FEC), study characteristics (design, randomization method, blinding, etc.). When extraction finished, data will be checked with each other by the two authors. Any dispute should be solved with the help of a third author. If the data is not complete, it is necessary to contact the original author. If data cannot be obtained still, we should transform the existing data or exclude the study.

### 2.5 Risk of bias assessment

Two authors will assess the methodological quality of all included studies independently based on the Cochrane Collaboration's tool.^[21]^ The following contents will be evaluated: random sequence generation, allocation concealment, blinding of participants and personnel, blinding of outcome assessment, incomplete outcome data, selective reporting and other biases. Each domain will be judged by the level of risk of bias: high level, low level, or unclear level. Any disagreements will be solved by discussion or with the help of a third author.

### 2.6 Data synthesis and statistical analysis

#### 2.6.1 Data synthesis

The software RevMan 5.3 will be used to construct the meta-analysis. To express the effects of physical therapy on lung function, it is necessary to extract the data change from the studies. The mean difference (MD) and the 95% confidence interval (CI) will be calculated for the continuous variable with a consistent unit. The standardized mean difference (SMD) and 95% CI will be calculated for the continuous variable with different units. *P* < .05 will be considered to be statistically significant.

#### 2.6.2 Assessment of heterogeneity

Heterogeneity will be assessed by the *χ*^2^ test and the *I*^2^ test. If *P* > .10 and *I*^2^ < 50%, the heterogeneity is acceptable and a fixed effect model will be used for data analysis. If *P* < .10 and *I*^2^ > 50%, we will search for the reasons for the high heterogeneity and use a random effects model for data analysis.

#### 2.6.3 Subgroup analysis

Subgroup analyses will investigate the effects of physical therapy according to the type of physical therapy (breathing exercises, inspiratory muscle training, and physical training), and the type of outcome measures on lung function (PEF, FEV1, FEC).

#### 2.6.4 Sensitivity analysis

Sensitivity analysis will be carried out based on the sample size, the missing data result and the methodological quality of the included study. If necessary, we will exclude a low-quality study and repeat the meta-analysis to test the stability of the pooled results.

### 2.7 Assessment of reporting bias

If more than 10 studied are included, a funnel plot will be used to examine the reporting bias. The results will be calcified based on the Cochrane Handbook for Systematic Reviews of Interventions.

### 2.8 Confidence in cumulative evidence

The quality of evidence will be assessed based on the Grading of Recommendations Assessment, Development, and Evaluation (GRADE) system. The evidence will be adjusted to 4 levels: high, moderate, low, or very low.

## 3 Discussion

Physical therapy is widely applied to improve lung function in children with asthma globally. However, the effects of physical therapy are still not certain, especially in children. To the best of our knowledge, the published review has examined the effects of physical training in people with asthma involving both children and adults, but not children only.^[22]^ Although some meta-analysis involved children only, they only examined the effects of one kind of physical therapies on children with asthma but not included all the 3 main therapies.^[23–25]^ Therefore, it is necessary to perform a systematic review and meta-analysis to investigate the effects of physical therapy on lung function in children with asthma and demonstrate which physical therapy is more effective. This study will be helpful to doctors treating asthma in children and provide some useful information when they are making the choice on which kind of physical therapy to be used.

However, this study will have some limitations. Language bias may exist because only studies published in English will be considered due to language barriers.

## Author contributions

Q Wang put forward the concept of this study. WJ Zhang drafted the preliminary version of this protocol. WJ Zhang and WH Yang will contribute to the study search, study selection, data extraction, and risk of bias assessment. WJ Zhang and LL Liu will complete the data analysis. Q Wang and HM Liu will help to solve any disagreement and ensure the quality of this study. Q Wang and WJ Zhang are co-first authors who contributed equally to this study. All authors critically reviewed, revised and approved the final manuscript.

**Conceptualization:** Qiu Wang.

**Data curation:** Weijian Zhang, Lilong Liu, Wenhao Yang.

**Methodology:** Weijian Zhang, Lilong Liu.

**Project administration:** Qiu Wang.

**Supervision:** Hanmin Liu.

**Writing – original draft:** Weijian Zhang.

**Writing – review & editing:** Qiu Wang, Hanmin Liu.
